# Early Weight Status and Human Capital in Adulthood: A 32-Year Follow-Up of the 1970 British Cohort Study

**DOI:** 10.3389/ijph.2024.1606829

**Published:** 2024-02-13

**Authors:** Yi Luo, Mimi Xiao

**Affiliations:** Research Center for Medical and Social Development, School of Public Health, Chongqing Medical University, Chongqing, China

**Keywords:** longitudinal study, obesity, human capital, weight, BCS70

## Abstract

**Objectives:** To prospectively evaluate the effects of early weight status (childhood and adolescence) and changes in obesity status on human capital in adulthood.

**Methods:** We employed data from the 1970 Birth Cohort Study in the United Kingdom. Data on height and weight during childhood and adolescence, human capital variables in adulthood were collected from 2,444 participants. Human capital includes cognitive ability, non-cognitive skill, educational attainment and health status. Data were analyzed through linear regression and logistic regression models.

**Results:** Our results showed that obesity during adolescence was negatively associated with cognitive ability (*β* = −0.83, *p* < 0.01), educational attainment (*β* = −0.49, *p* < 0.01), and some health outcomes; and that underweight in childhood also adversely affected educational attainment in females (*β* = −0.66, *p* < 0.05). In terms of changes in obesity status, becoming obese in adolescence negatively affected cognitive ability (*β* = −1.18, *p* < 0.01), educational attainment (*β* = −0.62, *p* < 0.05) and some health outcomes, remaining obese was associated with all adverse health outcomes.

**Conclusion:** Our results suggest that obesity during adolescence negatively affects a range of human capital outcomes in adulthood, and adolescence is a critical period during which early obesity affects adult human capital.

## Introduction

Over the past three decades, obesity has become one of the major public health problems in countries around the world [1]. The United Kingdom is one of the countries with the highest rates of childhood obesity, with recent figures showing that one in five children are obese. As we all know, overweight and obesity in children bring with them a host of social and health problems, such as low self-esteem, social discrimination, reduced quality of life, and the subsequent onset of a range of illnesses [2–4].

Evidence suggests that childhood obesity is linked to topics related to human capital development in the form of skill acquisition. For example, a study using a longitudinal dataset of Australian showed that children with obesity performed worse academically than normal children [5]. Human capital is defined as “the ability of humans to augment production potential through skill, knowledge, and effort” [6].

A number of studies have been conducted to show an association between early weight status and human capital [7–9]. We focused our research on obesity, research also shows that childhood obesity has a negative impact on children’s cognitive ability and non-cognitive skill [10–12]. A study conducted in the United States found that children who are obese have more problematic behaviours [10]. Another study conducted in Canada suggests that obesity is associated with impaired cognitive function in adults [12]. In contrast, there are also studies that did not find an association between early weight status and reading and math scores [13, 14]. Thus, overall, the relationship between early weight status and human capital does not point in a consistent direction. There are a variety of possible reasons for differences in the results of previous studies, including geographic and age variances in the study populations, the selection of control variables [13], and variations in the way human capital is measured [14]. Therefore, this suggests that we need further research to provide ample evidence.

Despite the existence of relevant studies [10–12], these studies still suffer from the following shortcomings: firstly, most of the studies investigating childhood obesity and human capital have been carried out at the same point in time, which prevents us from taking a long-term perspective on the early factors of human capital inequality and lacks an exploration of the impact on human capital in adulthood. Second, to the best of our knowledge, previous studies have defined human capital only as a single indicator of cognitive ability or educational attainment, rather than examining it as a multidimensional concept. Third, there have been reviews suggesting that adolescence may be a critical period for the impact of obesity on human capital [15, 16], but there is no evidence to argue this point.

Therefore, our study is based on a life course perspective to explore the impact of early weight status on human capital (as a multidimensional variable) in adulthood and to define whether adolescence is a critical time point for early obesity to affect human capital by tracking both childhood and adolescence.

## Methods

We used data from the 1970 British Cohort Study. An at-birth survey of over 17,000 people born between April 5 and April 11th, 1970 in England, Scotland, Wales, and Northern Ireland served as the foundation for the 1970 British Cohort Study (BCS70) [17]. The three major childhood surveys (ages 5, 10, and 16) include any children who were born outside of the country during the reference week but who were identified via school records at later ages, in addition to the initial birth cohort. In-depth data on the health and behaviour of the cohort members, as well as information about the family’s demographics and socioeconomic status, was gathered from parents and teachers in these childhood surveys. The six main adult surveys gathered data on employment, income, education, health, relationships, and attitudes from cohort members.

In the current analysis, Body Mass Index (BMI) and possible confounders were captured using data from birth to 16 years of age (1986), and outcome variables were evaluated at surveys conducted when study participants were 42 years of age (2012). Our analysis focuses on three stages: age 10 (childhood), age 16 (adolescence), and age 42 (adulthood). The reason for excluding other investigations is children do not have height and weight data at age 5 to calculate BMI, and other surveys of adulthood lack the variables in human capital that we wanted to study. Restricting the sample to members of the cohort present at ages 10, 16, and 42, only those cohort members with valid data on both BMI and human capital variables were included in the study. Finally, we are left with 2,444 cohort members as our sample.

### Weight Status Measurement

The body mass index (BMI) was employed as a surrogate measure for weight status, and was calculated for each age group by dividing weight in kilograms by height in meters (kg/m^2^). In the BCS70, children’s height and weight were obtained from a physical examination of the child by a community health officer. By calculating children’s BMI and comparing it to the BMI-for-age Percentile Growth Chart published by the Centers for Disease Control and Prevention (CDC). In our study, the percentage of children with early obesity was lower when defined by the 95th percentile, we therefore used the 85th percentile to measure early obesity status (include overweight and obesity); and underweight status if their BMI was below the 5th percentile. According to previous study, it is better to use overweight to measure the weight status of children and adolescents than to use obesity [18].

Weight status in children is a dynamic process, as the main variable we focused on was obesity, and one of the aims of our study was to explore whether adolescence is a critical period, we created categorical variables to capture differences in the effects of changes in obesity status on human capital. Children were defined as “never obese,” “remained obese” (obese in childhood and adolescence), “became obese” (Not obese in childhood, obese in adolescence) and “moved out of obesity” (obese in childhood, not obese in adolescence).

### Human Capital Measurement

It is becoming more widely accepted that, apart from education, other relevant characteristics—such as cognitive and non-cognitive skills and health are essential types of human capital since they produce income and other benefits as well [19]. In this study, the measurement of human capital in adulthood encompassed cognitive ability, non-cognitive skill, educational attainment and health status.

Cognitive ability in adulthood was assessed using vocabulary task in this study [17]. There were 20 terms in the vocabulary task, and five more words were listed next to each of them. Cohort members were asked to choose which of the five words that were placed next to each of the 20 words had the same meaning to the original word. Cohort members were given a time limit of 4 minutes to complete the task. The maximum number of characters correctly reported by the respondent will be used as their cognitive ability score (0–20).

As non-cognitive assessments, we employed survey items related to the Big Five personality traits. Adult personality traits have been demonstrated to be highly stable [20]. The Big Five personality theory divides personality into five fundamental characteristics: openness to experience, conscientiousness, extroversion, agreeableness, and emotional stability [21]. We used five items for the Big Five personality measures. Specifically, the item “I’ve been interested in new things” was used to measure openness to experience; the item “I’ve been dealing with problems well” was used to measure conscientiousness; the item “I’ve been feeling confident” was used to measure extraversion; the item “I’ve been feeling close to other people” was used to measure agreeableness. The item “I’ve been feeling optimistic about the future” was used to measure emotional stability. The answers to each question ranged from none of the time to all of the time, the corresponding score ranges from 1 to 5. The overall big five personality index is calculated by summing scores for the five main traits and dividing by 5, with a higher rating indicating greater non-cognitive skill.

Educational attainment in adulthood was measured using the highest qualification obtained (age 42) in this study. We divided the academic qualification into six levels: (1) No qualification; (2) Lower-level school qualification (bad O-levels, Certification of Secondary Education grades 2–5); (3) Middle level school qualification (good O-levels, 2+ AS levels or 1 A level); (4) Higher level school qualification (more than one A level); (5) First degree (diploma, degree); (6) Higher degree. Therefore, educational attainment in this study was scored on a scale of 0 (no qualification) to 5 (higher degree).

Health status in adulthood in this study included four dimensions: poor general health; diabetes; BMI >30; high blood pressure. Cohort members were asked to rate their general health on a 5-point scale representing excellent, very good, good, fair and poor health. This is dichotomised into 0 for excellent/very good/good health and 1 for fair/poor health. Diabetes was obtained through the question “Have you had diabetes mellitus since the last interview,” yes = 1, no = 0. BMI was calculated using self-reported height and weight at age 42. High blood pressure was obtained through the question “Have you had high blood pressure since the last interview,” yes = 1, no = 0.

### Covariates

We account for a large number of variables that may confound the association between early weight status and human capital. Birth weight (as a continuous variable), maternal education, household income, family receiving supplementary benefits, and father’s social class were included as covariates in the current analyses. Because 99.14% of the sample in our study was England, so we did not include race in the covariate to explore. Specifically, mother’s education was evaluated when the cohort member was 10 years old and was coded (0) for “no qualifications” or [1] for “some qualifications.” Family receive supplementary benefits (yes = 1, no = 2). Household income (weekly) is divided into seven levels and assigned a value ranging from 1 to 7, and the higher the value, the higher the income. The Registrar General’s measure of social class was used to assess father social class, it is scored on a 6-point scale with “professional” being assigned a score of 1 and “unskilled” receiving a score of 6. The scoring was reversed so that a high score denoted a high social position.

### Statistical Analysis

Multiple linear regression models were developed to quantify the effects of early weight status and changes in obesity status on cognitive ability, non-cognitive skill and educational attainment. Since health status is a categorical variable, the odds ratios (ORs) were employed in conjunction with 95% confidence intervals (CIs), which were calculated using logistic regression, to summarize the impact of early weight status and changes in obesity status on health capital in adulthood.

Previous researches have shown that the impact of obesity on human capital differs between boys and girls [10, 11, 14]. Given its importance, we used either subgroup regressions by gender or gender interaction terms to analyze for all endpoints in this study, and all findings were adjusted for our earlier covariates. As a birth cohort study, age control was not necessary. All analyses were performed using Stata Statistical Software, release V.17.

## Results

### Sample Characteristics

Table 1 gives a description of the data we used in this study. 1,313 (53.72%) of the sample were female; about 4.13% of the households received supplementary benefits; 56.83% of them had a mother with a qualification certificate; the mean birth weight was 3.37 kg; the weekly household income was divided into 7 levels and the mean weekly income of the sample households was 3.96; father’s social class was divided into 6 levels with mean values of 3.57. In addition, 224 (9.17%) and 127 (5.20%) were obese and underweight at age 10; and 283 (11.58%) and 127 (5.20%) were obese and underweight at age 16. The state of obesity in children is also dynamic, and in our sample, with 168 (6.87%), 109 (4.46%), and 115 (4.71%) individuals become obese, moved out of obesity, and remained obese from age 10–16 years, respectively. As for the outcome variables of the study, cognitive ability scores with a mean of 13.46; non-cognitive skill with a mean of 3.54; educational attainment with a mean of 4.09. Besides, there were 275 people (11.25%) with poor general health, 52 (2.13%) with diabetes, 151 (6.18%) with high blood pressure, and the amount of people with BMI >30 at age 42 was 517 (21.15%).

**TABLE 1:** Descriptive results of participants. Early weight status and human capital in adulthood: a 32-year follow-up of the 1970 British Cohort study, United Kingdom, 2023.

Variables	*N*	Percent	Mean	SD
Female	1,313	53.72		
Birth weight			3.37	0.49
Family receive supplement benefits	101	4.13		
Family income (per week)			3.96	1.46
Mother has some qualifications	1,389	56.83		
Father’s social class (1–6)			3.57	1.43
Weight status				
Obesity, age10	224	9.17		
Underweight, age10	127	5.20		
Obesity, age16	283	11.58		
Underweight, age16	127	5.20		
Change in obesity status				
Became obese	168	6.87		
Moved out of obesity	109	4.46		
Remained obese	115	4.71		
Human capital, age 42				
Cognitive ability			13.46	3.31
Non-cognitive skill			3.54	0.64
Educational attainment			4.09	2.75
Health status				
Poor general health	275	11.25		
Diabetes	52	2.13		
High blood pressure	151	6.18		
BMI>30	517	21.15		

In Table 2, we analyze the effect of early weight status on human capital at age 42. After controlling for all confounders, the results showed that across all populations, obesity in adolescence was significantly and negatively associated with cognitive ability (*β* = −0.83, *p* < 0.01) and educational attainment (*β* = −0.49, *p* < 0.01). Besides, underweight in childhood also had a negative impact on educational attainment (*β* = −0.51, *p* < 0.05). For non-cognitive skill, we did not find an association between early weight status and non-cognitive skill. In stratified analyses by gender, obesity in adolescence negatively affected both cognitive ability and educational attainment, which was consistent across males and females. Negative effects of childhood underweight on educational attainment found only in females (*β* = −0.66, *p* < 0.05). Table 3 showed the results of changes in obesity status, we found that only becoming obese in adolescence demonstrated significant negative effects on both cognitive ability (*β* = −1.18, *p* < 0.01) and educational attainment (*β* = −0.62, *p* < 0.05) in adulthood, which did not differ between males and females (*p* > 0.05, gender*type of change in obesity status).

**TABLE 2:** Relationship between early weight status and skills aspects of human capital. Early weight status and human capital in adulthood: a 32-year follow-up of the 1970 British Cohort study, United Kingdom, 2023.

Variables	Cognitive	Non-cognitive	Educational attainment
Entire sample			
Obesity, age10	0.47* (0.24)	0.02 (0.05)	0.22 (0.20)
Underweight, age10	−0.18 (0.30)	0.02 (0.06)	−0.51** (0.24)
Obesity, age16	−0.83*** (0.22)	0.01 (0.04)	−0.49*** (0.18)
Underweight, age16	0.01 (0.30)	−0.01 (0.06)	0.43* (0.25)
Male			
Obesity, age10	0.58 (0.36)	−0.07 (0.07)	0.18 (0.30)
Underweight, age10	−0.17 (0.43)	−0.04 (0.08)	−0.32 (0.36)
Obesity, age16	−1.10*** (0.34)	0.09 (0.07)	−0.61** (0.28)
Underweight, age16	−0.48 (0.40)	−0.08 (0.08)	0.41 (0.34)
Female			
Obesity, age10	0.36 (0.33)	0.11 (0.07)	0.27 (0.27)
Underweight, age10	−0.19 (0.41)	0.07 (0.08)	−0.66** (0.33)
Obesity, age16	−0.62** (0.29)	−0.06 (0.06)	−0.44* (0.23)
Underweight, age16	0.58 (0.44)	0.09 (0.09)	0.44 (0.36)

Standard errors in parentheses. *p < 0.1, **p < 0.05, ***p < 0.01.

**TABLE 3:** Relationship between changes in obesity status and skills aspects of human capital. Early weight status and human capital in adulthood: a 32-year follow-up of the 1970 British Cohort study, United Kingdom, 2023.

Variables	Cognitive	*p* (interation)	Non-cognitive	*p* (interation)	Educational attainment	*p* (interation)
became obese	−1.18***	0.40	0.12	0.07	−0.62**	0.57
	(0.39)		(0.08)		(0.32)	
moved out of obesity	0.43	0.71	−0.04	0.26	0.18	0.64
	(0.44)		(0.09)		(0.36)	
remained obese	−0.33	0.76	0.01	0.75	−0.52	0.53
	(0.46)		(0.09)		(0.38)	
*N*	2,444		2,444		2,444	
F	22.62		2.73		23.13	
Adjusted *R* ^2^	0.10		0.01		0.10	

Standard errors in parentheses. *p < 0.1, **p < 0.05, ***p < 0.01.

**FIGURE 1:**
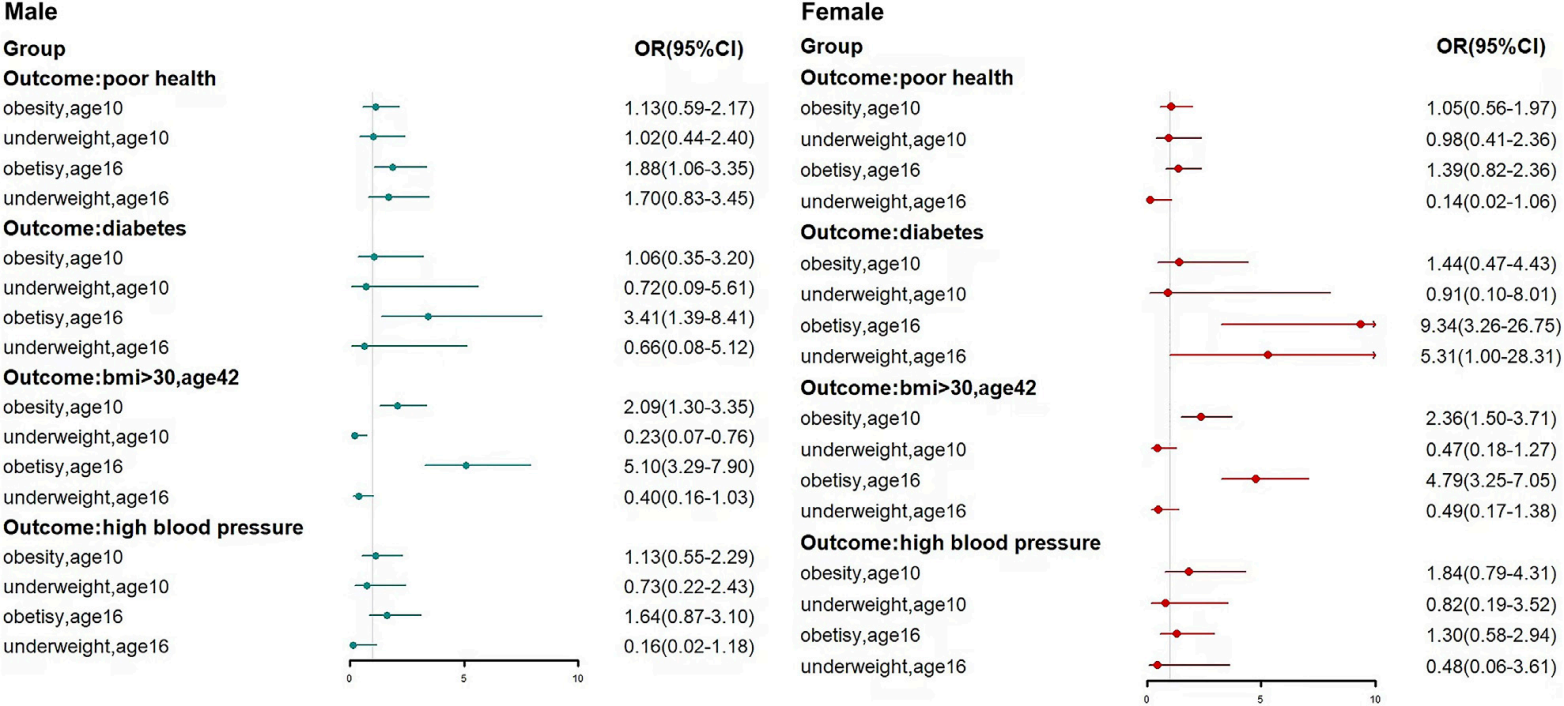
Forest plots of the relationship between early weight status and health capital. Early weight status and human capital in adulthood: a 32-year follow-up of the 1970 British Cohort study, United Kingdom, 2023.

**FIGURE 2:**
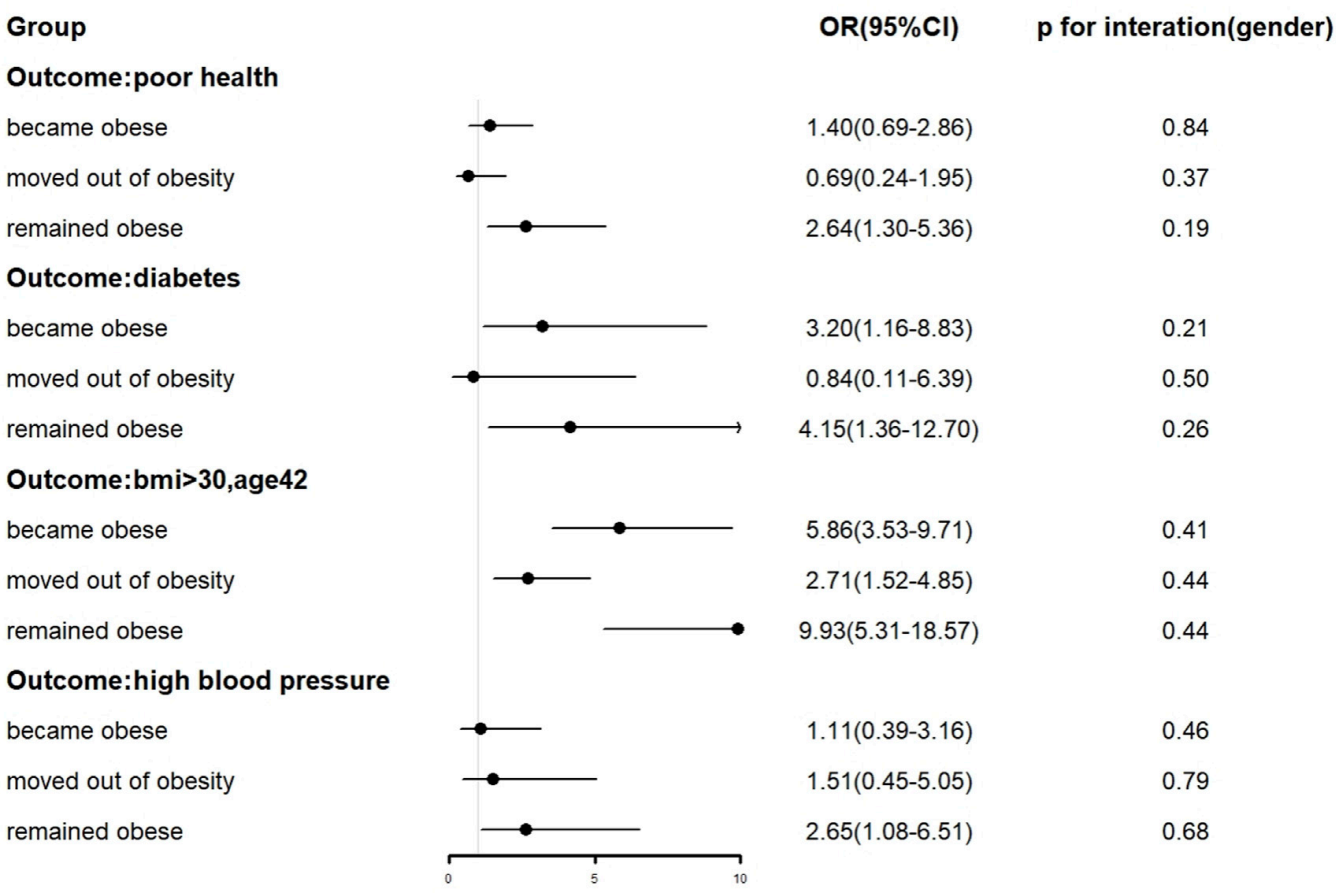
Forest plot of the relationship between changes in obesity status and health capital. Early weight status and human capital in adulthood: a 32-year follow-up of the 1970 British Cohort study, United Kingdom, 2023.

Stratified by gender to analyze the relationship between early weight status and health capital in adulthood, the results are presented in a graphical format (Figure 1). After adjusting for all confounder, the results showed that obesity in adolescence was associated with diabetes in adulthood for both males (OR = 3.41, 95% CI: 1.39–8.41) and females (OR = 9.34, 95% CI: 3.26–26.75), and that both obesity in childhood and obesity in adolescence led to a significantly increased risk of BMI >30 in adulthood. Figure 2 showed the impact of changes in obesity status on health capital, we found that remaining obese and becoming obese during adolescence both led to a significant increase in the risk of diabetes (OR = 4.15, 95% CI: 1.36–12.70; OR = 3.20, 95% CI: 1.16–8.83) and BMI>30 (OR = 9.93, 95% CI: 5.31–18.57; OR = 5.86, 95% CI: 3.53–9.71) in adulthood. And remaining obese was also associated with poor health (OR = 2.64, 95% CI: 1.30–5.36) and high blood pressure (OR = 2.65, 95% CI: 1.08–6.51). These findings did not differ between males and females (*p* > 0.1, gender*type of change in obesity status).

## Discussion

In the nationally representative British birth cohorts, the aim of our study is to investigate the impact of early weight status on human capital in adulthood. Because our primary weight status of interest is obesity, the results can deepen the understanding of the long-term human capital inequalities associated with early obesity. To the best of our knowledge, this is the first study to investigate the impact of early weight status on adult human capital. Our study shows that obesity during adolescence has a significant negative effect on human capital in adulthood. We argue that adolescence is a critical period in which early obesity affects human capital.

Our results are consistent with evidence from samples drawn from the United States of America [22] and Australia [5]. Specifically, in the current study, obesity in adolescence was significantly and negatively associated with cognitive ability in adulthood, which is similar with previous studies [7–9]. Our study differs in that other studies have examined children’s cognitive performance, while we take a “life course” perspective on adult outcomes. Several previous reviews have suggested that children with overweight/obesity measured at older ages are more likely to suffer negative effects, which provides support for our study [15, 16]. The reason could be that adolescent obesity is a strong indicator of adult obesity [23, 24], which may have a negative impact on cognition due to connections with vascular disease [25]. Besides, overweight/obesity causes individuals to have reduced cardiorespiratory fitness, and studies have shown that early cardiorespiratory fitness has a significant impact on cognitive performance in midlife [25, 26].

Furthermore, our results show that obesity during adolescence has a negative effect on educational attainment in adulthood, which is consistent with existing research [27]. It’s worth noting that we’ve found that for female, being underweight also has a negative impact on educational attainment, which received support from existing relevant studies [28–30]. The reason may be that obesity, as well as underweight, is susceptible to discrimination and stigmatization compared to those children of normal weight, thus affecting their educational development [31, 32]. Another possible explanation is that depression mediates the relationship between obesity and lower educational attainment [27]. Our findings support the view that the impact of obesity on educational attainment is not only significant among females but also among males, and even more so for males, which is consistent with existing research [27]. We need to recognize this to avoid exacerbating further gender inequalities.

In the analyses of changes in obesity status, we found that in terms of skills in human capital, only becoming obese in adolescence showed significant negative effects with cognitive ability and educational attainment. This is a direct indication that adolescence is a critical period when obesity affects the skill aspects of human capital in adulthood. To our knowledge, our study is the first to empirically identify adolescence as the critical period during which obesity affects human capital in adulthood, which can be explained by the critical period hypothesis. The critical period hypothesis suggests that impacts are more likely to be experienced at a particular point in childhood (i.e., around the age of 12), arguing that certain stages in people’s lives will have a specific impact on the rest of their lives [33]. Therefore, attention should be paid to the critical juncture of adolescence when developing obesity interventions in order to prevent its long-lasting consequences. In previous review exploring the relationship between obesity and human capital, it was noted that researchers are encouraged to conduct cohort studies to assess whether different associations exist based on whether obesity is measured in childhood, adolescence, or adulthood [15]. And our study further provides evidence and support for this direction.

The effects of early obesity on health in adulthood are obvious, and some researches have been done to explain this [34, 35]. We focus our discussion on the relationship between changes in obesity status and health. First, remaining obese was significantly and negatively associated with all adverse health outcomes (poor health, diabetes, BMI >30, and high blood pressure). Secondly, becoming obese also significantly increases the risk of diabetes and BMI >30 in adulthood. We argue that these findings can be explained by the theory of cumulative disadvantage, which is consistent with existing research [36]. Cumulative disadvantage theory accentuates that early strengths or weaknesses are critical for groups to become differentiated over time [37]. However, previous study did not include childhood and adolescence, so our study is an extension and addition to it. The earlier the impact of weight on health is recognized, the earlier timely interventions can be provided. Early risk factors have an impact on both short- and long-term consequences. Risk factor effects build up over the course of a lifetime, thus increasing heterogeneity in later life [38, 39].

### Strengths and Limitations

This study has some strengths. First, our longitudinal study design using birth cohorts, grounded in the life course to explore the impact of early weight status on human capital in adulthood. And longitudinal studies can be effective in mitigating reverse causality to some extent. Second, the BMI we used to measure weight status was measured objectively rather than self-reported, and the prospective measurement of BMI in early life as opposed to distant recall in midlife. Third, we have a wider range of results on measures of human capital in our study, which is unusual in the context of the existing literature, allowing us to examine human capital more comprehensively. Fourthly, our study tracked weight status during childhood and adolescence, thus classifying changes in obesity status to validate the key period during which obesity affects human capital. This enables governments, communities and families to adopt timely intervention strategies during a critical period to prevent the long-lasting harms of early obesity.

Our study is of course not without several limitations. Firstly, we used BMI calculated from height and weight to measure early weight status. Obesity is caused by excess body adiposity, but BMI does not take into account the distribution of adiposity, and in addition to height and weight, the amount of adiposity obtained by using the triceps skin folds can be more accurately defined as the obesity status of a child [40]. The second limitation is that the results may not be generalizable to other, more diverse populations, as participants were recruited only from the England, Scotland, Wales, and Northern Ireland, and the response rate to the follow-up survey was low. Another limitation is that measures of non-cognitive skills and health status in adulthood are obtained by self-report, which makes it prone to reporting bias. Therefore more researches are needed to expand further.

### Implications

Our study highlights that obesity during adolescence can have long-lasting effects on cognitive skills and educational achievement in adulthood. Analyses of changes in weight status also further suggest that adolescence is a critical period for early obesity to influence human capital in adulthood. This requires us to realise that the damage to human capital caused by early obesity does not stop at the current point in time, but has a long-lasting negative impact. The critical juncture of adolescence should therefore be taken into account in the development of interventions targeting obesity. Families and communities should focus on weight status in early childhood to prevent the long-term disadvantages that early obesity brings to children.

For future studies, we suggest using the amount of fat gained from triceps skin folds as an alternative to BMI to measure childhood obesity, which could more accurately capture obesity in children. In addition, future research can further investigate the intermediate pathway through which obesity affects human capital to help us clarify the mechanism.

### Conclusion

The results of our analyses suggest that early obesity has an important negative impact on the content of human capital in adulthood to suggest that many effects extend further into the adult life course than previously understood. Findings also suggest that adolescence being a key period in which obesity affects human capital, which may have important implications for the direction and timing of future policy and practice interventions.
